# A bacterial effector cleaves RIN4 to allow the dimerization and activation of its recognizing NLR

**DOI:** 10.1093/plphys/kiae555

**Published:** 2024-10-18

**Authors:** Manuel González-Fuente

**Affiliations:** Assistant Features Editor, Plant Physiology, American Society of Plant Biologists; Faculty of Biology & Biotechnology, Ruhr-University Bochum, 44780 Bochum, Germany

The vascular pathogen *Ralstonia solanacearum*, responsible for bacterial wilts on more than 250 different plant species, is one of the most devastating pathogens due to its aggressiveness, large and expanding geographical distribution, and long-lasting persistence in water and soil (Landry et al. 2020). Like many other pathogens, *R. solanacearum* relies on the translocation of effector proteins into the host cell to subvert the host defenses and accommodate pathogen needs (Arroyo-Velez et al. 2020). In turn, some plants have evolved Nucleotide-binding Leucine-rich Repeat receptors (NLRs) that recognize some of these effectors and trigger strong immune responses such as cell death that limits pathogen proliferation (Ngou et al. 2022). *Ralstonia* has one of the largest repertoires of effectors among bacteria, many of which trigger immune responses in different hosts (Landry et al. 2020). However, to date, only 3 NLRs that recognize *R. solanacearum* effectors have been described. One of these is the solanaceous Ptr1 (Pseudomonas tomato race 1), which in addition to recognizing *R. solanacearum* effector RipBN (Ralstonia injected protein BN) (Mazo-Molina et al. 2019) also recognizes 5 effectors from other pathogenic bacteria (Ahn et al. 2023). The discovery and molecular characterization of such immune receptors will be key to develop novel and durable resistance strategies against pathogens whose virulence and distribution are alarmingly increasing in the current context of global warming.

**Figure 1.**
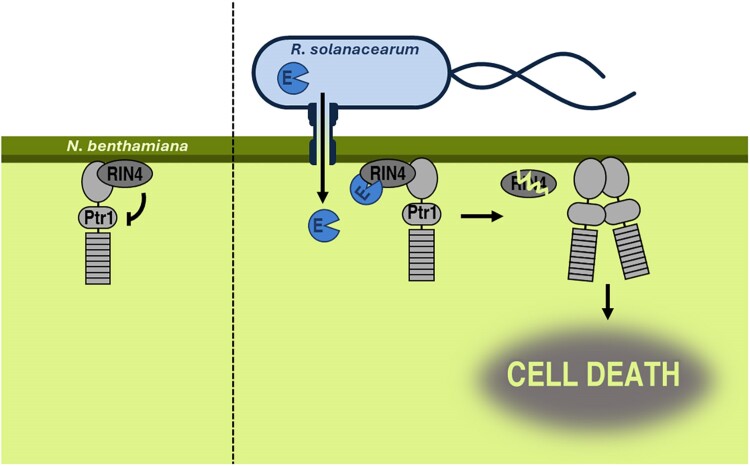
Bacterial effector RipE1 cleaves RIN4 to derepress Ptr1-mediated cell death. In the absence of the pathogen, plasma membrane–localized RIN4 binds to the coiled-coiled domain of the immune receptor Ptr1 preventing its dimerization and activation. Upon infection, *Ralstonia solanacearum* effector protein RipE1 (E in the figure) is translocated into the host cell where it proteolytically degrades RIN4, releasing Ptr1 that can now dimerize through its coiled-coiled domain and activate its downstream signaling to ultimately cause immunogenic cell death that prevents the colonization of the bacteria.

In this issue of *Plant Physiology*, Lu et al. (2024) dissect the molecular mechanism behind the Ptr1-mediated recognition of the conserved *R. solanacearum* effector RipE1 (Ralstonia injected protein E1) in *Nicotiana benthamiana* via proteolytic degradation of the negative immune regulator RIN4 (RPM1 INTERACTING PROTEIN 4). The authors genetically corroborated that the previously reported cell death caused by RipE1 was mediated by Ptr1 (Jeon et al. 2020; Kim et al. 2023). This NLR was already known to be negatively regulated by the “effector hub” RIN4 (González-Fuente et al. 2020; Ahn et al. 2023). This inspired Lu and colleagues to study whether the Ptr1-mediated recognition of RipE1 was also regulated by *N. benthamiana* RIN4. Indeed, the authors showed that cysteine protease RipE1 interacted with and cleaved RIN4, similar to other effector proteases (Axtell et al. 2003; Mazo-Molina et al. 2019). This cleavage of RIN4 was required for RipE1/Prt1-mediated cell death, as neither the catalytic dead mutant of RipE1 nor the cleavage-insensitive mutant of RIN4 could induce cell death. Moreover, the authors also discovered that in basal conditions, RIN4 specifically interacts and prevents the self-association of the coiled-coiled (CC) domain of Ptr1. The dimerization of the NLR CC domains is important for their downstream signaling (Maekawa et al. 2011). Therefore, the cleavage of RIN4 by the bacterial protease RipE1 releases Ptr1, allowing Ptr1 to homodimerize and activate the immune signaling that ultimately causes cell death (Fig. 1).

The authors also corroborated that the *N. benthamiana* Ptr1 can recognize effectors from different pathogenic bacteria. Complementarily, they also showed that *R. solanacearum* RipE1 is recognized by Ptr1 orthologs from several solanaceous species. This, together with the wide conservation of RIN4 among land plants, reveals the potential of the Ptr1/RIN4 regulatory hub in generating broad-spectrum resistance. Interestingly, the *Pseudomonas* ortholog of RipE1, HopX1, does not seem to be recognized by *N. benthamiana* Ptr1. This provides an excellent chance for comparative and structural studies that would shed more light into the domains and motifs specifically required for effector recognition and immune signaling, paving the way to engineer more efficient and broader-spectrum immune receptors in the future. Moreover, the authors also showed that RipE1 protease activity is required not only for its immune recognition but also for its virulence function. This expands the catalogue of bacterial effectors whose enzymatic activity is required for both virulence and avirulence (Leong et al. 2022; Lauber et al. 2024).
